# Phenylketonuria patients’ and their parents’ acceptance of the disease: multi-centre study

**DOI:** 10.1007/s11136-016-1326-2

**Published:** 2016-05-31

**Authors:** Ewa Witalis, Bożena Mikoluc, Radoslaw Motkowski, Justyna Szyszko, Agnieszka Chrobot, Bozena Didycz, Agata Lange, Renata Mozrzymas, Andrzej Milanowski, Maria Nowacka, Mariola Piotrowska-Depta, Hanna Romanowska, Ewa Starostecka, Jolanta Wierzba, Magdalena Skorniewska, Barbara Iwona Wojcicka-Bartlomiejczyk, Maria Gizewska, Polish Society of Phenylketonuria

**Affiliations:** 1 Medical and Rehabilitation Centre in Gdansk, Gdansk, Poland; 2 Department of Pediatrics and Developmental Disorders of Children and Adolescents, Medical University of Bialystok, ul. Waszyngtona 17, 15-274 Bialystok, Poland; 3 Brudzinski Children’s Hospital in Bydgoszcz, Bydgoszcz, Poland; 4 Polish-American Institute of Pediatrics, Cracow, Poland; 5 Polish Mother’s Memorial Hospital, Lodz, Poland; 6 Department of Pediatrics, Regional Specialist Hospital, Research and Development Centre, Wrocław, Poland; 7 National Institute of Mother and Child, Warsaw, Poland; 8 Department of Pediatrics, Endocrinology, Diabetology, Metabolic Disorders and Cardiology, Pomeranian Medical University, Szczecin, Poland; 9 Department of Pediatrics, Hematology and Oncology, Medical University of Gdansk, Gdansk, Poland; 10 Medical Department Nutricia, Warsaw, Poland

**Keywords:** Phenylketonuria, Children, Parents, Disease acceptance

## Abstract

**Purpose:**

Phenylketonuria (PKU) still poses a therapeutic challenge for patients and medical professionals. The aim of the study was to assess both patients’ and their parents’ acceptance of the disease.

**Methods:**

The study included 218 PKU patients and 178 parents of PKU children who were enrolled in the study on the basis of questionnaire data.

**Results:**

Regarding attitude towards the disease, our study demonstrated that 63 (28.9 %) PKU patients did not accept the disease. Patients who found accepting the disease difficult, more frequently perceived themselves as inferior/different in comparison with their peers. In total, 36 % of patients did not want their friends to be aware of their condition, while only 18 % of parents believed that their children’s peers should not know about their disease. In total, 42 % of parents wanted to talk to other parents of PKU children and only 13 % to a doctor. Only 20 % of patients saw the need to discuss their condition with a doctor. In total, 8 % of children, regardless of age, and 14 % of parents preferred to talk to a psychologist.

**Conclusion:**

Our data demonstrated that disease acceptance played an essential role in patients’ social integration. The study also indicated the need to overcome communication barriers between patients and their healthy peers and for patients to find the courage to be open about the disease. The importance of support groups for PKU families and the significance of strict cooperation between patients and their families with PKU treatment teams were also revealed.

## Introduction

Phenylketonuria (PKU OMIM:261600), first recognised by Dr Asbjørn Følling in 1934 and considered a prime example of predictive medicine, still poses a therapeutic challenge for both patients and medical professionals. Although the dietary management currently recommended enables PKU patients to achieve full personal and social development, for parents of an affected child, the diagnosis means living in a state of constant anxiety. The anxiety, which results from the necessity of adhering to the rules imposed by the disease, is often present from the first days of the child’s life.

The disease forces, initially, parents and subsequently patients to learn the principles of nutritional management which includes a daily meticulous control of food intake and a strict adherence to the prescribed health regimen. This means that from early infancy, the implications of the condition shape the social and psychological aspects of PKU patients’ family life. Therefore, a subjective assessment of the quality of life by PKU patients and their families is undoubtedly one of the crucial factors which influences the effectiveness of their therapy. In the case of children, it is the behaviour of adults and their attitude to the disease that affects the outcome of dietary management and the children’s acceptance of the disease.

In 1993 *The* *World Health Organization* and *The* *International Association for Child Psychology and Psychiatry* recommended self-assessment as the most appropriate method to measure children’s quality of life. Hence, information on the assessment of PKU patients’ and their families’ quality of life should enable the introduction of measures which would facilitate the effective implementation of dietary management standards and improve the families’ quality of life.

The aim of the study was to assess both patients’ and their parents’ acceptance of the disease and to analyse these attitudes in different age groups. An attempt was made to determine how disease acceptance affected patients’ self-assessment and interpersonal relationships both within the family and in a peer group.

## Patients

The study included 218 individuals with classic PKU (108 females and 110 males) aged 10–35, managed in 9 specialist metabolic clinics in Poland. The PKU patients were enrolled in the study on the basis of questionnaire results. The following inclusion criteria were applied: PKU diagnosed in early infancy in a newborn screening test (at follow-up tests blood PHE concentration >20 mg/dl), exclusion of atypical forms of PKU (loading tests with phenylalanine and tetrahydrobiopterin, urinary pterin profiles), a low phenylalanine diet from early infancy, intellectual development within age norm (children within the public education system).

Individuals suffering from mild PKU and other chronic diseases were excluded from the study. Characteristics of the PKU patients are presented in Table 1.

**Table 1:** Characteristics of PKU patients (*n* = 218)

Age at study (years)	*N*	%	Male		Female	
			*N*	%	*N*	%
10–13	70	32.11	38	54.29	32	45.71
14–16	69	31.65	30	43.48	39	56.52
17–19	34	15.60	18	52.94	16	47.06
≥20	45	20.64	24	53.33	21	46.67
Total	218		110	50.46	108	49.54

The study also included 178 parents of PKU children (132 mothers, 46 fathers). Over 70 % of them described their family’s economic conditions as very good or good, 25 % as average and 1 % as poor. More than 70 % of parents had vocational or secondary education, 25 % had higher education and 4 % had primary school education.

## Methods

The study used a questionnaire designed by the research team in cooperation with clinical psychologists assisting PKU patients and their families, and experts from the Polish Society of Phenylketonuria. The questionnaire completed by the study participants has been attached.

The study was conducted during regional and national meetings of PKU families with therapists. Participation in the survey was voluntary. Members of PKU families not participating in the meetings were not approached and asked to take part in the study. The questionnaire contained 9 questions on the patients’ and their parents’ attitudes to the disease. The questions concerned the acceptance of the disease, self-perception in the context of the disease, social relations and communication needs in the family and the community. Questions for parents and children were similar in format. A Likert-type response scale was used in the survey.

The family’s socio-economic status was only assessed by the parents. Children below the age of 15 completed the questionnaire either unassisted or with the help of a qualified researcher.

### Statistical analysis

All calculations were performed using the Microsoft Excel spreadsheet and STATISTICA, StatSoft, Inc., version 8.0 statistical package (data analysis software system). Statistical evaluation of quantitative data utilised the classical measures of location such as arithmetic means and medians, and measures of variation such as standard deviation and range. Normality distribution of the variables and variance equality of studied features in groups was established with the use of Shapiro–Wilk test and variance equality test. In order to compare groups in pairs for quantitative data, *t* test or Mann–Whitney test were used with respect to the type of distribution of the variables tested. In the case of multiple group comparison, the Kruskal–Wallis test was used as the nonparametric equivalent of one-way analysis of variance (ANOVA). In all these calculations, the statistical significance level was set at *p* < 0.05.

## Results

**Fig. 1:**
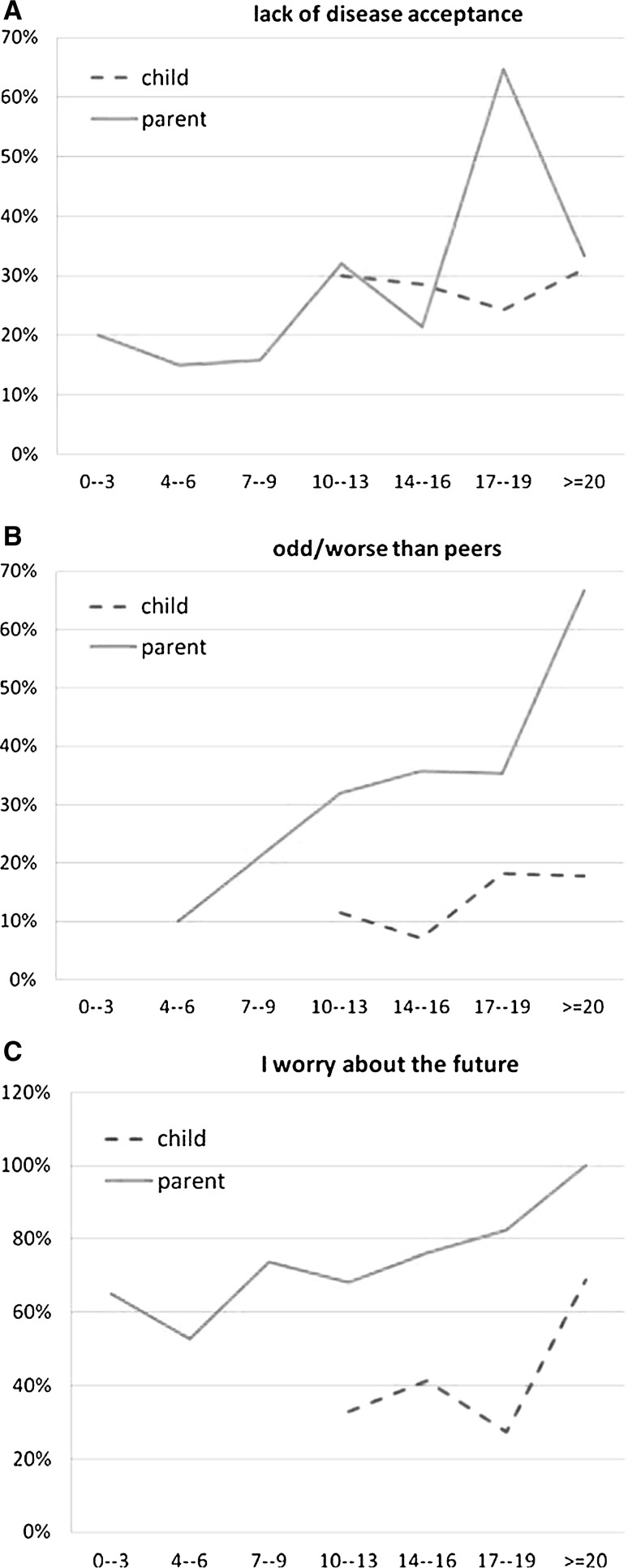
Disease acceptance (**a**), self- perception (**b**), disease acceptance and perception of educational and future professional career in PKU patients (**c**)

Regarding attitude towards the disease, our study demonstrated that in the group of 218 PKU patients, 63 (29 %) did not acknowledge their disease. Furthermore, out of the 178 parents surveyed, 49 (28 %) displayed a conspicuous lack of acceptance of their children’s disease (Figure 1a).

It was observed that parental lack of acceptance increased when the child was between the ages of 7–19 (*p* = 0.0111). In regard to disease acceptance by children, no differences were found between age groups (*p* = 0.6817).

Only 12 % of the children surveyed stated that they perceived themselves as inferior/different from their peers. However, twice as many parents (28 %) believed that their children felt inferior to their peers because of the disease. The conviction strengthened as the child grew older (Fig. 1b).

**Fig. 2:**
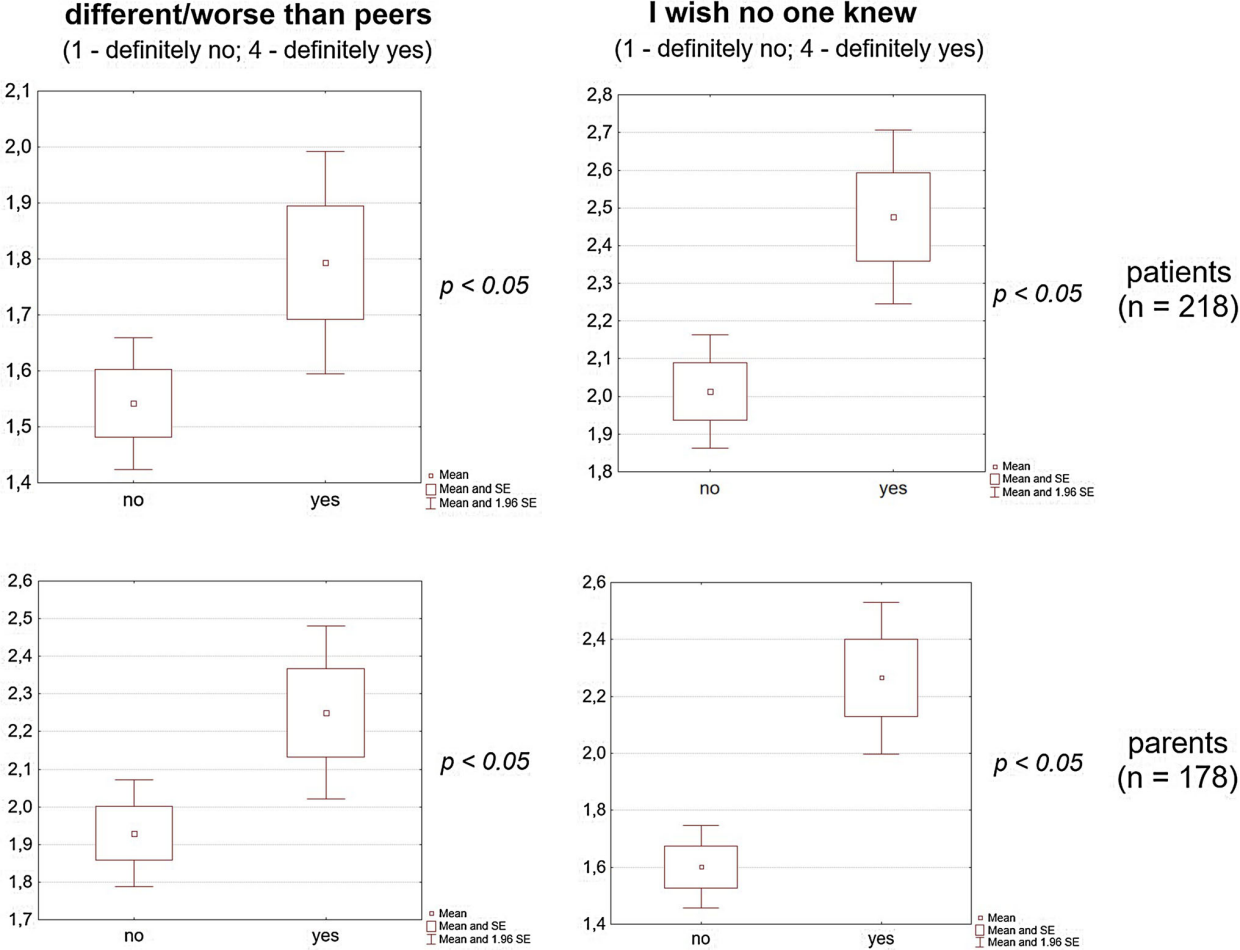
Disease acceptance in PKU parents, patients and self-assessment in relation to their peers

Irrespective of age, patients who found accepting the disease difficult were more likely to perceive themselves as inferior/different in comparison with their peers (*p* = 0.0173) and preferred not to discuss their condition with them (*p* = 0.0008). Similarly, parents who experienced difficulty in accepting their child’s disease were more inclined to believe that their child felt inferior/different from his/her peers (*p* = 0.0236) and were more likely to prefer their peers not to know about their child’s condition (*p* = 0.0001) (Fig. 2).

The study demonstrated that 64 % of the patients surveyed wanted to share knowledge of their disease with their friends, expecting their support and acceptance (59 % of PKU parents). On the other hand, 36 % of patients did not want their friends to be aware of their condition, while only 18 % of parents believed that their children’s healthy peers should not know about their disease.

### Disease acceptance, perception of educational and future professional career

The study revealed that 71 % of parents experienced anxiety about their children’s future, which increased with the child’s age, while only 42 % of patients were concerned about their future (Fig. 1c).

**Fig. 3:**
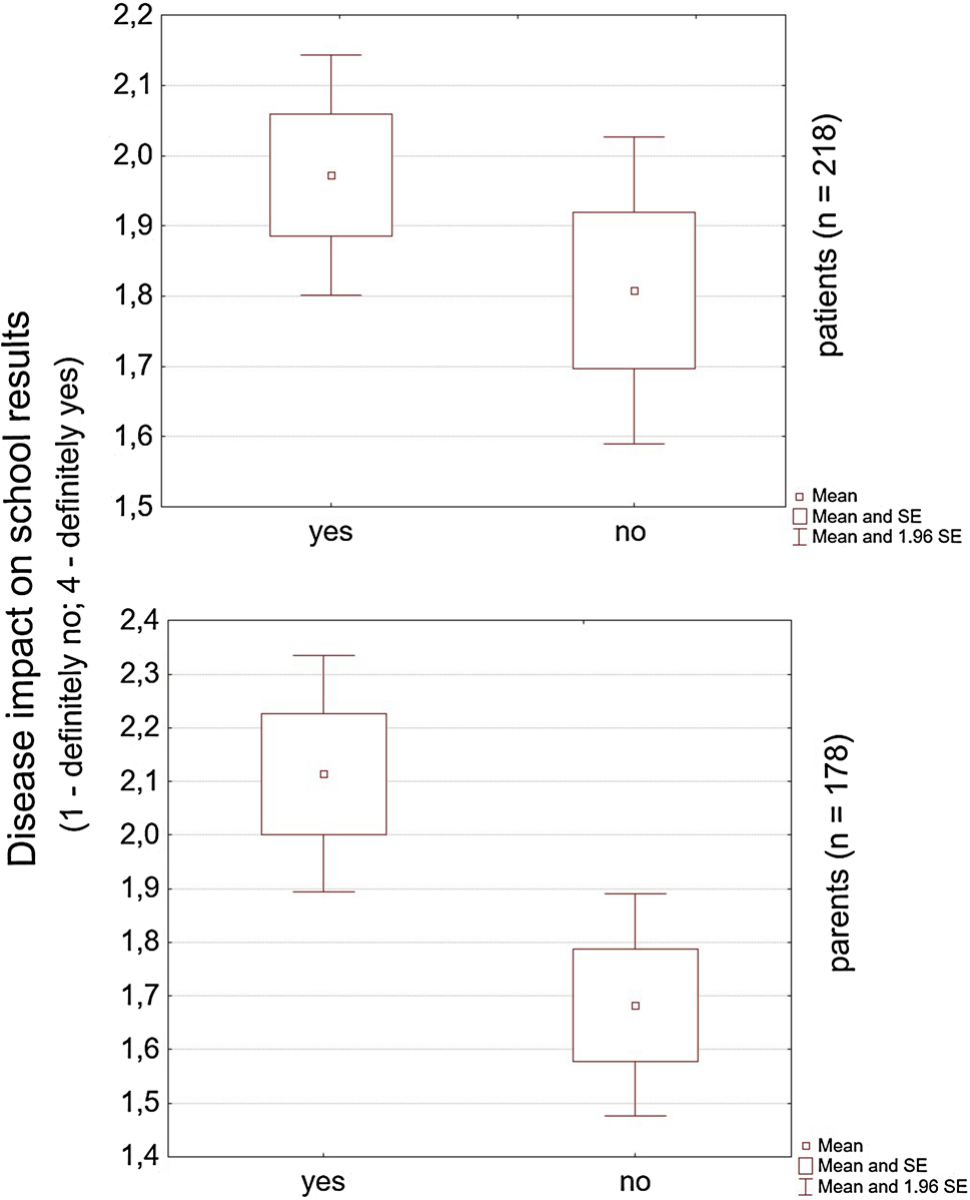
Disease acceptance in PKU patients and their parents on school results

The study demonstrated that 66 % of children believed that PKU affected their education, which was especially evident in patients over the age of thirteen. 50 % of parents shared this opinion. Both the parents and patients, irrespective of age, expressed a strong conviction that the disease impacted on school results if it was not accepted (*p* = 0.1722) (*p* = 0.0022) (Fig. 3).

### Discussing the disease—seeking and receiving support

The study demonstrated that 96 % of parents believed that they devoted a sufficient amount of time to discussing the disease with their children, while only 63 % of children shared this opinion.

**Fig. 4:**
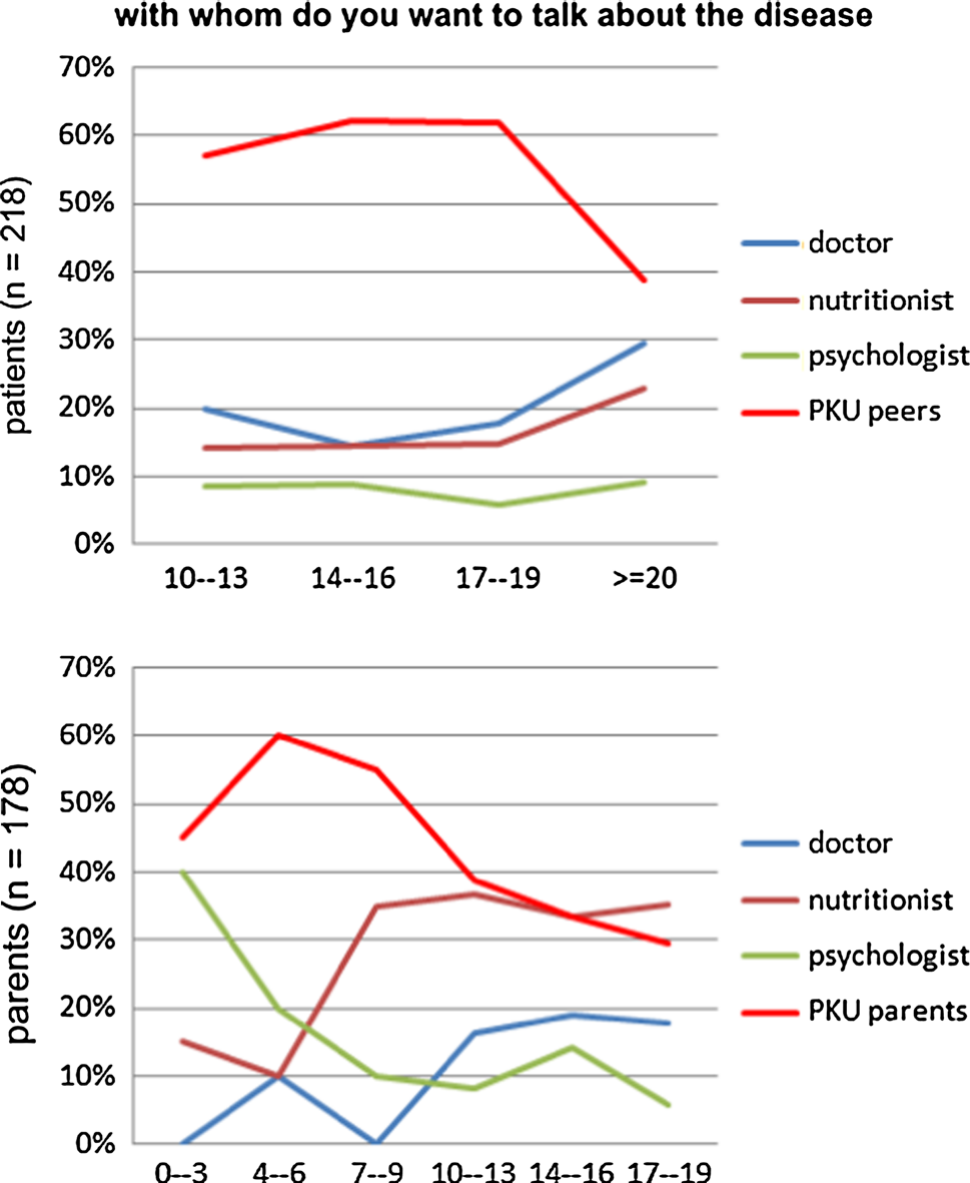
Discussing the disease—seeking and receiving support

When given the choice of discussing their condition with a professional (a doctor, a nutritionist, a psychologist) or a peer suffering from the same disease, more than half of PKU patients (56 %) named peers. On the other hand, 42 % of parents declared that they wanted to talk to other parents of PKU children and only 13 % wanted to talk to a doctor. It is remarkable that although both the parents and children consider the doctor to be the main source of information about the disease, only 20 % of patients saw the need to discuss their condition with a doctor. Only approximately 8 % of children, regardless of age, preferred to talk to a psychologist. It is worth mentioning that in the first 3 years of the child’s life, 40 % of parents preferred to consult a psychologist. The number fell to 20 % in the following three years (children aged 4–6) and further decreased to 10 % when the children reached the age of 7 (Fig. 4).

We would like to emphasise the fact that the study did not show a correlation between blood Phe concentration levels in the year prior to the study and the responses provided by the patients and their parents. Blood tests demonstrated that Phe levels were higher than recommended in the group of patients above the age of 14.

## Discussion

Analysis of the questionnaire results on PKU patients’ and their parents’ attitudes towards the disease revealed that the majority of children (71 %) and their parents accepted the condition, while 25 % of the respondents claimed that they would never accept it. The obtained results are consistent with a study by Lord et al. [1] which demonstrated that for a number of parents the stress associated with a PKU diagnosis may be an extremely traumatising event whose impact can last even until the child reaches the age of 12. The daily nutritional management and regular monitoring of blood Phe levels can be a constant reminder of the presence of a potential danger. This may explain parents’ lack of disease acceptance even years after the diagnosis [2]. Our study revealed similar results in both parents and adult patients. Additionally, we found that a lack of disease acceptance increased in parents of teenagers (17–19), while it was not age related in patients themselves.

The study demonstrated that non-acknowledgement of the disease had a negative effect on interpersonal relationships and on self-perception in the context of a peer group. Children who did not accept their condition did not want others to be aware of it and felt inferior/different from their healthy peers. Nonetheless, they simultaneously believed that life would be easier for them if all their friends knew about their disease.

Similarly, Di Ciommo et al. [3] noted that the main problem of PKU patients was their self-perception in the context of their age group when they compared themselves to their peers, which was frequently accompanied by a fear of being stigmatised. Young people with PKU often experience problems in explaining their health condition and dietary needs to healthy individuals [4]. This confirms a need for overcoming communication barriers between PKU children and healthy peers and underlines the importance of a patient’s ability to inform others about the disease.

Our study demonstrated that professional assistance should be provided to prepare PKU patients to talk openly about their condition. It is important to teach patients adequate interpersonal communication skills as well as ways to verbalise their needs. Educating parents is equally important since our study revealed that parents who found accepting their child’s condition difficult preferred them not to discuss their disease with healthy peers.

The study demonstrated that the degree of disease acceptance affected the patients’ perception of their educational abilities and a future career. We found that lack of such acceptance made PKU patients more inclined to see a negative impact of the condition on their academic achievement and to be more concerned about their future career prospects when compared with patients who accepted the disease. The results of the study confirmed that disease acceptance was a critical factor influencing PKU patients’ quality of life.

Similar issues concerning disease acceptance have been demonstrated in patients with other chronic diseases such as cystic fibrosis, diabetes or neurodegenerative diseases. The problem of disease acceptance in adolescents and young adults with cystic fibrosis and diabetes was presented by Casier et al. [5], according to whom illness acceptance was positively related to patients’ general well-being. Increased levels of social isolation, depressive mood and anxiety were revealed by research studies involving teenagers with diabetes and phenylketonuria [6–8]. A study by Brumm et al. [9] demonstrated low self-esteem, decreased social competence, a greater likelihood of school problems, a lower level of motivation and decreased autonomy. A study by Simon on socio-demographic status of adolescents and young adults with PKU revealed a tendency towards a lower degree or delayed autonomy as well as a lower rate of building normal adult relationships or having children [10]. As the studies conducted to date demonstrate, the problem of accepting the disease poses a challenge for the patient, his or her parents, peer groups as well as medical professionals. Therefore, it is crucial that steps to improve the level of disease acceptance by PKU patients and their parents are taken. At this stage of our research, however, we have not been able to establish the reasons for disease non-acceptance in such a high percentage of respondents.

An important element of the conducted study was an attempt to investigate how parents perceived their PKU children in the context of their peer group. Our research demonstrated that parents were more likely to believe that their children felt inferior to their healthy peers. In fact, the number of parents making this claim was twice as high as that of children. Despite such a perception of their children’s situation, the parents were convinced that the children should inform their healthy peers about their condition. The obtained results suggest that parents are not aware of the fact that patients prefer their peers not to know about their medical problems. Parents also do not realise that their children often choose to withdraw from their age group, which can result in a lack of close relationships. Discussing the disease also seems to pose a problem for many patients. As our study indicates, parents are usually unaware of their children’s need to discuss their condition specifically with them. In general, parents believe that they have adequately satisfied this need at every stage of their child’s life.

Therefore, it would be advisable for medical professionals to devote particular attention to the scope of communication needs within the family in an attempt to improve the current status.

The study also explored the patients’ and their parents’ need to discuss the disease with professionals and individuals affected by the disease. The results demonstrated that both the children and their parents wanted to talk about the condition to people in a similar situation. There was a strong need among the patients to talk to their affected peers, while the parents surveyed expressed a desire to discuss the issue with other families of PKU children. Surprisingly, medical professionals involved in PKU patient management were not selected as preferred discussion partners. Despite the fact that all the respondents regard the doctor to be the main source of information on phenylketonuria, they stated a preference for discussing the condition with a group of people sharing a similar experience. Only 17 % of those surveyed stated that they preferred to talk to a doctor about the disease, which may suggest that the need for medical consultation and information about phenylketonuria has been met in both patients and their parents.

Our study confirmed the need for promoting age-related and ‘expert patient’-type interventions, especially to help individuals suffering from chronic conditions [11–14]. Other research has shown that offering support to people in a similar situation can be of benefit to everyone involved and assisting others can induce increased feelings of self-esteem [11], reduce depressive symptoms [15] and institute changes in one’s self-management [16].

Our study also demonstrated that the desire expressed by parents of children under the age of 3 to consult a psychologist was as strong as that for talking to other parents of PKU patients. The study revealed that parents of the youngest children (under 6 years of age) felt the greatest need for psychological support.

The aforementioned results confirm the benefit of establishing support groups for parents of PKU children as well as providing them with professional psychological care. It is also worth noting that for parents of children under the age of 9, apart from a doctor, a nutritionist constitutes an important source of information about the disease. In the course of our study, we observed that the parents’ need to talk to a nutritionist increased after the child’s 7th birthday and was, at that time, three times stronger than the need to consult a psychologist. Among the parents of school-age children, the need to consult a nutritionist increased in subsequent years.

Our findings support the conclusion reached by other authors that a nutritionist plays a significant role not only in the therapeutic process but also in assisting parents with the daily adherence to the PKU diet [17–19]. Our results are consistent with other research data which confirm the important role of a nutritionist in the management of patients with PKU and other chronic diseases [20–23].

The analysis of the responses obtained from the adult patients surveyed revealed surprising results. It became evident that, in comparison with the earlier periods of the respondents’ lives, both negative self-assessment (patients’ sense of inferiority/difference) and an unfavourable perception of professional development prospects strengthen. It is clear, however, that despite lower self-esteem adult patients do not see the need for a psychological consultation. It was also noted that those patients have different communication needs regarding their peers and medical professionals. The need to maintain relationships with people in a similar situation is strongest for PKU patients under the age of 19, while for patients over the age of 20 this need diminishes, and those patients express a preference to discuss their problems with medical professionals. The majority of adult patients want to talk to a doctor and a nutritionist, while the need for a psychological consultation remains at an unchanged, low level.

The study suggests that adult PKU patients express more critical opinions about themselves and their future personal and professional prospects, and their needs for discussing the disease are different from younger patients’.

## Conclusion

The study, conducted on a large group of PKU patients and their parents, demonstrated that disease acceptance played an essential role in the patients’ social integration and their perception of a future professional career. It is therefore vitally important to make a concerted effort to facilitate an appropriate attitude towards the disease in the family and to improve parent–child communication. The study also revealed the need to overcome communication barriers between patients and their healthy peers and for patients to find the courage to be open about the disease. The importance of support groups for PKU families and of strict cooperation between patients and their families with PKU treatment teams was also revealed.

Further research is needed to fully understand the reasons for lack of disease acceptance in both patients and their parents.

## References

[1] Lord B, Wastell C, Ungerer J (2005). Parent Reactions to Childhood Phenylketonuria. Families, Systems, and Health.

[2] Lord B, Ungerer J, Wastell C (2008). Implications of resolving the diagnosis of PKU for parents and children. Journal of Pediatric Psychology.

[3] Di Ciommo V, Forcella E, Cotugno G (2012). Living with phenylketonuria from the point of view of children, adolescents, and young adults: a qualitative study. Journal of Developmental and Behavioral Pediatrics.

[4] Sharman R, Mulgrew K, Katsikitis M (2013). Qualitative analysis of factors affecting adherence to the phenylketonuria diet in adolescents. Clinical Nurse Specialist.

[5] Casier A, Goubert L, Gebhardt WA, Baets FD, Aken SV, Matthys D, Crombez G (2013). Acceptance, well-being and goals in adolescents with chronic illness: a daily process analysis. Psychology Health.

[6] Weglage J, Funders B, Ullrich K, Rupp A, Schmidt E (1996). Psychosocial aspects in phenylketonuria. European Journal of Pediatrics.

[7] Weglage J, Funders B, Wilken B, Schubert D, Schmidt E, Burgard P, Ullrich K (1992). Psychological and social findings in adolescents with phenylketonuria. European Journal of Pediatrics.

[8] Weglage J, Grenzebach M, Pietsch M, Feldmann R, Linnenbank R, Denecke J, Koch HG (2000). Behavioural and emotional problems in early-treated adolescents with phenylketonuria in comparison with diabetic patients and healthy controls. Journal of Inherited Metabolic Disease.

[9] Brumm VL, Bilder D, Waisbren SE (2010). Psychiatric symptoms and disorders in phenylketonuria. Molecular Genetics and Metabolism.

[10] Simon E, Schwarz M, Roos J, Dragano N, Geraedts M, Siegrist J, Kamp G, Wendel U (2008). Evaluation of quality of life and description of the sociodemographic state in adolescent and young adult patients with phenylketonuria (PKU). Health and Quality Life Outcomes.

[11] Barlow JH, Bancroft GV, Turner AP (2005). Volunteer, lay tutors’ experiences of the Chronic Disease Self-Management Course: being valued and adding value. Health Education Research.

[12] Bodenheimer T (2003). Primary care in the United States. Innovations in primary care in the United States. BMJ.

[13] Dennis CL (2003). Peer support within a health care context: a concept analysis. International Journal of Nursing Studies.

[14] Nicholas DB, Keilty K (2007). An evaluation of dyadic peer support for caregiving parents of children with chronic lung disease requiring technology assistance. Social Work in Health Care.

[15] Pfeiffer PN, Heisler M, Piette JD, Rogers MA, Valenstein M (2011). Efficacy of peer support interventions for depression: a meta-analysis. General Hospital Psychiatry.

[16] Kingsnorth S, Gall C, Beayni S, Rigby P (2011). Parents as transition experts? Qualitative findings from a pilot parent-led peer support group. Child: Care, Health and Development.

[17] Bilginsoy C, Waitzman N, Leonard CO, Ernst SL (2005). Living with phenylketonuria: perspectives of patients and their families. Journal of Inherited Metabolic Disease.

[18] MacDonald A, Asplin D (2006). Phenylketonuria: practical dietary management. The Journal of Family Health Care.

[19] Singh RH, Rohr F, Frazier D, Cunningham A, Mofidi S, Ogata B, Splett PL, Moseley K, Huntington K, Acosta PB, Vockley J, Van Calcar SC (2014). Recommendations for the nutrition management of phenylalanine hydroxylase deficiency. Genetics in Medicine.

[20] Bernstein LE, Helm JR, Rocha JC, Almeida MF, Feillet F, Link RM, Gizewska M (2014). Nutrition education tools used in phenylketonuria: clinician, parent and patient perspectives from three international surveys. Journal of Human Nutrition and Dietetics.

[21] Franz MJ, Monk A, Barry B, McClain K, Weaver T, Cooper N, Upham P, Bergenstal R, Mazze RS (1995). Effectiveness of medical nutrition therapy provided by dietitians in the management of non-insulin-dependent diabetes mellitus: a randomized, controlled clinical trial. Journal of the American Dietetic Association.

[22] Jennings HC, Connolly CB, Lamance K, Dominguez B (1999). Phenylketonuria (pku) camp promotes dietary compliance and reduction in serum phenylalanine (phe) levels. Journal of the American Dietetic Association.

[23] Singh RH, Kable JA, Guerrero NV, Sullivan KM, Elsas LJ (2000). Impact of a camp experience on phenylalanine levels, knowledge, attitudes, and health beliefs relevant to nutrition management of phenylketonuria in adolescent girls. Journal of the American Dietetic Association.

